# PCSK9 signaling pathways and their potential importance in clinical practice

**DOI:** 10.1007/s13167-017-0106-6

**Published:** 2017-08-14

**Authors:** Michał Wiciński, Jarosław Żak, Bartosz Malinowski, Gabriela Popek, Grzegorz Grześk

**Affiliations:** Department of Pharmacology and Therapeutics, Faculty of Medicine, Collegium, Medicum in Bydgoszcz, Nicolaus Copernicus University, 85-090 Bydgoszcz, Poland

**Keywords:** PCSK9, LDL, Metabolism, Cell signaling, Inflammation, Apoptosis

## Abstract

In the following review, authors described the structure and biochemical pathways of PCSK9, its involvement in LDL metabolism, as well as significances of proprotein convertase subtilisin/kexin type 9 targeted treatment. PCSK9 is a proprotein convertase, which plays a crucial role in LDL receptor metabolism. Transcription and translation of PCSK9 is controlled by different nuclear factors, such as, SREBP and HNF1α. This review focuses on interactions between PCSK9 and LDL receptor, VLDLR, ApoER2, CD36, CD81, and others. The role of PCSK9 in the inflammatory process is presented and its influence on cytokine profile (IL-1, IL-6, IL-10, TNF) in atherosclerotic plaque. Cholesterol metabolism converges also with diabetes by mTORC1 pathways. PCSK9 can be altered by oncologic pathways with utilization of kinases, such as Akt, JNK, and JAK/STAT. Finally, the article shows that blocking PCSK9 has proapoptotic capabilities. Administration of monoclonal antibodies against PCSK9 reduced mortality rate and cardiovascular events in randomized trials. On the other hand, immunogenicity of new drugs may play a crucial role in their efficiency. Bococizumab ended its career following SPIRE-1,2 outcome. PCSK9 inhibitors have enormous potential, which had been reflected by introducing them (as a new class of drugs reducing LDL concentration cholesterol) into New Lipid Guidelines from Rome 2016. Discoveries in drugs development are focused on blocking PCSK9 on different levels. For example, silencing messenger RNA (mRNA of PCSK9) is a new alternative against hypercholesterolemia. Peptides mimicking EGF-A domain of the LDL receptor are gaining significance and hopefully they will soon join others. The significance of PCSK9 has just been uncovered and further data is still required to understand their activity.

## Background

PCSK9 inhibitors are a new class of drugs that bring great hope for the treatment of hypercholesterolemia. Their importance was noticed 14 years ago. It all started with the discovery of a third gene related to familial hypercholesterolemia, which is responsible for the synthesis of connexin 9 proprotein. PCSK9 gene is located in the vicinity of the gene for LDL receptor and ApoB on the short arm of the first chromosome (1p32.3). The triad of genes for PCSK9, ApoB, and LDL receptor plays a key role in cholesterol metabolism.

Inside the human body, cholesterol is transported in combination with lipoproteins. Lipoproteins are biochemical assemblies, which role is to transport lipids in extracellular fluid. We can distinguish several classes of plasma lipoproteins such as: HDL (high-density lipoproteins), LDL (low-density lipoprotein), IDL (intermediate-density lipoprotein), VLDL (very low-density lipoprotein), and chylomicrons. Density and size of lipoproteins depend on the fat/protein ratio. HDL particles are the smallest and contain the biggest amount of protein, mostly ApoA. Their function is to collect cholesterol, triglycerides, and phospholipids from different tissues and transport them to the liver. LDL particles are responsible for transport of fats to different tissues. Their main apolipoprotein is ApoB100, which is a ligand for activation LDL receptor (LDL R). Accumulation of cholesterol carrying by LDL in arteries wall leads to atherosclerosis and to cardiovascular diseases such as stroke or myocardial infarction.

LDL is absorbed into the cell after combining with LDL receptor on the surface of the hepatocyte. LDL receptor degradation depends on the level of PCSK9. This cholesterol circuit is controlled by various factors, which require further research.

## Structure and function of PCSK9

Regulation of biochemical changes takes place at different levels. Many of the synthesized proteins remain in an inactive form. Only the interactions with mediators reveal their true meaning. Proteins of the convertase proprotein (PC) group constitute enzyme modulators. They include the following: PC1, PC2, Furin, PC4, PC5, PACE4, PC7, and isoenzyme 1 subtilisin kexin and PCSK9. These enzymes by their catalytic centers cleave the precursor proteins, hormones, growth factors, receptors, and various transmembrane proteins [1, 2]. The only exception is PCSK9, which reveals its action by self-activation.

PCSK9 is 74 kDa zymogen consisting of 692 amino acids. The enzyme is composed of a signaling peptide (aa 1–30), prodomain (aa 31–152), catalytic domain (aa 153–404), hinge region (aa 405–454), and the C-terminal domain rich in cysteine and histidine (CHRD) (aa 452–692) [3]. In the latter one, three modules have been distinguished: M1 (aa 453–531), M2 (aa 530–605), and M3 (aa 604–692). The module richest in histidine proved to be M2 containing 9 out of 14 histidines from across the domain (CHRD) [4, 5]. A mature form of PCSK9 consisting of 60 kDa and a 14 kDa N-terminal prodomain is created in the endoplasmic reticulum. This process involves autocatalytic cleavage at position FAQ152 [6]. Despite the division of the original form of PCSK9, they are still connected by non-covalent binding between the prodomain and catalytic domain and a C-terminal [7]. In this way, the prodomain allosterically blocks the action of the other two domains. Experimental removal of the prodomain results in a ten times higher affinity of PCSK9 for LDLR and a four times higher rate of degradation of the LDLR [8, 9]. A high frequency of mutations (34%) of this region in human demonstrates the prodomain importance for the proper activity of PCSK9 [10]. Prodomain is therefore a factor that modulates the effect of PCSK9 by releasing the catalytic domain.

The active form of PCSK9 combines with EGF domain-A LDL receptor. The intracellular pathway PCSK9 facilitates the transport of LDLR from the Golgi apparatus to lysosomes. Clathrin light chains participate in this process. The study found that blocking the synthesis of clathrin light chains increases the number of LDL receptors, regardless of the activity of extracellular PCSK9 [11, 12]. In the extracellular pathway, after being released from the Golgi apparatus, a mature form of PCSK9 is internalized together with LDLR in endosomes and undergoes the process of degradation in lysosomes. The internalization of PCSK9-LDLR complex in endosomes may take place only in the presence of cytoplasmic adapter (ARH—low-density lipoprotein receptor adapter protein) [13]. Mature 60 kDa form of PCSK9 can be cleaved at 218 RFHR position by other proproteins, i.e., furin and PC5/ 6A, leading to the formation of a 53-kDa form, which is ten times less active compared to LDLR. It was found that in humans, this form may constitute up to 15–40% of the circulating forms of PCSK9 [14, 15].

## Regulation of transcription PCSK9

The synthesis of PCSK9 is controlled at the transcriptional level by the SREBP-2. SREBP-2 is a membrane transcription factor which regulates the conversion of PCSK9. It connects to the conservative transcription motif SRE (serum response factor) occurring in the promoter of PCSK9 and LDLR gene. The SREBP family consists of two isoforms. SREBP-2 affects LDL and PCSK9 receptor genes. SREBP-2 is involved in the synthesis and absorption of cholesterol by affecting the gene expression of inhibitor reductase HMG CoA and synthase HMG CoA. The second member of the transcription factors family SREBP-1c is involved in fatty acid synthesis. It regulates transcription of fatty acid synthase and carboxylase alpha and beta acetyl-coenzyme A. SREBP-1 and SREBP-2 connect to the promoter of (SRE) PCSK9 and LDLR gene. The state of hunger or satiety is a decisive factor in SREBP activation. In the case of deficiency of intracellular cholesterol, cholesterol sensor (SCAP) enables the transport of SREBP-2 to the Golgi apparatus, where its proteolytic maturation takes place. As a result of this process, having entered the nucleus, a mature form of SREBP-2 can interact with the promoter (SRE-1 region) of PCSK9 and LDLR leading to an increased transcription and translation of both proteins [16–18].

In one of the studies, a 36-h period of famine in hamsters caused a PSCK9 decrease by 60%, with a comparable reduction in the expression of mRNA PCSK9. That research also determined a 50% reduction in the expression of mRNA SREBP and, as a consequence, a reduction in lipogenesis genes controlled by them. A drop of 75% decrease of HNF1α was determined in animal livers [19]. Similar results were obtained treating rats with glucagon. Postprandial satiety state balanced by the state of hyperinsulinemia caused the return of selected parameters to the state before the period of fasting. In contrast to the clear correlation occurring between PCSK9 and SREBP-1 in the experiment, there was no similar interrelation between levels of LDLR and SREB-2 [20]. Thus, the state of fasting influences the expression of PCSK9 and LDLR in a different way. The daily rhythm of eating and fasting reflects the existing relationship between the level of PSCK9 and LDLR. The Dubac et al.’s research administering statins that block cholesterol synthesis triggered the activation of SREBP2, which resulted in increased transcription of PCSK9 mRNA and, consequently, increased LDLR turnover [21].

Another nuclear factor affecting the expression of PCSK9 is HNF1α (hepatocyte nuclear factor 1 homeobox A), which is located near the SRE motif. It is found in primates and rodents. Its presence has not been found on the gene promoter for LDLR. Its inhibition or stimulation affected proportionally the expression of the PCSK9, modifying LDL-C levels. Statins also stimulated the expression of HNF1α leading to increased production of PCSK9 [22, 23].

## PCSK9 and HDL

Previous papers have shown a negligible effect of PSCK9 on HDL level. Until now, no direct interactions of PCSK9 with VLDL and chylomicrons have been discovered. On the other hand however, it has been noted that the higher the level of VLDL, the greater the ability of PCSK9 to form di- and trimers—structures of a far greater ability to degrade LDLR. PCSK9 catalytic domain is responsible for the formation of these multimolecular forms of PCSK9. The reverse principle in the construction of PCSK9 oligomers is related to HDL particles. The higher their level is, the fewer the PCSK9 oligomers, the more LDL receptors, and the higher the turnover [24]. It can be concluded from the presented relations that HDL can significantly create lipid balance by influencing the activity of PCSK9.

On the other hand, PCSK9 inhibitors in recent experiments and clinical trials within phases II and III showed little influence on HDL level and on the associated ApoA1. OSLER study found a mere 7% increase in HDL and a 4.2% increase of ApoA-1 after the administration of ewolocumab [25]. Meanwhile, alirocumab in the ODYSSEY long-term study caused an increase of HDL and ApoA-1 by 4.6 and 2.9%, respectively [26]. In animal models, the usage of antisense oligonucleotides against PCSK9 reduced HDL-C level in mice by 54% and administration of antibodies against PCSK9 reduced this level by 30% [27, 28].

Differences in this study were brought about by different metabolic pathways in rodents and primates. In humans, CETP (cholesteryl ester transfer protein) is responsible for the transport of triglycerides and cholesterol esters between HDL and LDL and VLDL, which contain Apo B. CETP is absent in rodents [29]. CETP inhibitors in the second phase of clinical studies resulted in a HDL increase of 179.1% and 63.4% of ApoA-1, and an LDL-C decrease of 45.3% and Apo B of 33.7% [30]. Studies with new CETP inhibitors such as anacetrapib and K-312 demonstrated a SREB1- and SREB2-dependent reduced level of PCSK9. The reduction of PCSK9 was independent of CETP in that study [31, 32].

## PCSK9 versus other receptors

PCSK9 demonstrates an activity towards the family members of the receptor for LDL, ApoER2, and VLDLR. PCSK9 ability to degrade LRP1 (low-density lipoprotein receptor-related protein 1) has not been proven in vivo yet. ApoER2 receptor exhibits 46% homology of similarity in the amino acid sequence with LDLR and 59% with VLDLR. In Poirier’s research, degradation of both particles was independent of PCSK9 catalytic center, as well as, of LDLR presence. In a reversed experiment, it was found that the activity of PCSK9 towards LDLR is independent of the presence of the other two receptors [33, 34]. Research shows that VLDLR and LDLR but not ApoER2 compete for the degradation by PCSK9, with VLDLR being more susceptible to the influence of PCSK9.

ApoER2 and VLDLR receptors are located in the cerebellum and are involved in the early stages of brain development. Lack of expression of both receptors in mice caused neurodegenerative changes in the cerebral cortex and cerebellum [35].

PCSK9 also interacts with CD36 receptor, which among other, is responsible for facilitating the absorption of free fatty acids in the intestinal epithelial cells and adipocytes [36, 37]. PCSK9 also affected the degradation of NPC1L (Niemann-Pick C1-like 1 protein)—a cholesterol transporter whose blocking is the mechanism of ezetimibe action—cholesterol lowering drug by about 20%. In CD36 and CD81 receptors, no EGF-A binding domain was found; thus, their interaction with PCSK9 is subject to other regulations.

The most common side effects of administering PCSK9 inhibitors are infections of the upper respiratory tract. It is possible that the increased LDLR turnover facilitating the penetration of viruses into the body is responsible for this status quo. Labonte’s study determined that the level of CD81 receptor (HCV) is regulated by PCSK9. HCV virus particles bind with VLDL and LDL. Viral entry into cells is mediated by CD81 and LDLR and also by NPC1L [38]. PCSK9 inhibitors, despite their unprecedented advantages in the reduction of LDL, can cause increased penetration of HCV into hepatocytes. Will ezetimibe be the appropriate drug for the treatment of hypercholesterolemia in patients with HCV? This hypothesis requires further research.

## PCSK9 pathways in inflammatory and diabetes

PCSK9 can affect the normal functioning of pancreas. Mice lacking the gene fprPCSK9 had higher expression of LDLR. They displayed low insulin levels and high glucose levels. Their insulin resistance was associated with irregularities in the pancreatic islet construction. Deposition of cholesterol in β cells resulted in severe inflammation and a tendency for faster apoptosis [39].

The metabolic pathways of cholesterol cross with diabetes pathways. Miao et al. in his study found a direct LDLR degradation correlation with insulin-stimulated increase of PCSK9 expression. In experiments on mice where insulin receptor were deactivated by the use of antisense oligonucleotides (ASO) or streptozotocin, the level of PCSK9 dropped to 80%. Interestingly, a similar reduction in the level of PCSK9 was noted in mice starved with locked insulin receptors (LIRKO—liver insulin receptor knockout). This phenomenon can be explained by the existence of additional mechanisms influencing PCSK9.

It is possible that glucagon comes into play here as in experiments on rats that it reduced the level of PCSK9 mRNA by 50%. In addition, glucagon reduced the level of transcripts SREBP-1c and SREBP-2 from 20 to 50%, whereas the level of Pck1 expression increased nine times. In that study, glucagon caused a 20% decrease in the level of LDLR mRNA but most importantly, a doubled level of LDL receptors was found on the surface of hepatocytes [40].

In another study, observation in the early stage of diabetes and metabolic syndrome characterized by high level of VLDL, low HDL level, and constant level of LDL led to the conclusion that high LDL turnover is responsible for this cholesterol fraction, dependent on hyperinsulinemia, as in patients with full length diabetes type 2, wherein the insulin level is low and LDL had slower metabolism. Similarly, experiments on mice with obesity in hyperinsulinemia stage revealed a decreased level of PCSK9 mRNA by 60%, with an increased level of the number of LDL receptors. This study led to the identification of a new pathway regulating the level of PCSK9. It turned out that insulin stimulates a complex (mTORC1), which by activation of PKCδ inhibits the activity of HNF4α and HNF1α, and in this way, also the transcription of PCSK9 [41]. MTORC1 pathway is therefore the link between lipid metabolism and diabetes.

These findings explain hypercholesterolemia occurring in patients treated with an immunosuppressant drug, rapamycin (kinase mTOR inhibitor), after kidney transplantation. PCSK9 inhibitors may become a targeted anti-cholesterol therapy in humans using various generations of mTORC1 inhibitors in not only immunosuppressant treatment but also in anti-cancerogenic one. The mTORC1 complex is the mediator of many metabolic cycles. New oncology drugs focus on its blocking, bringing very encouraging results. It is possible that in the future, an individualized approach to the oncology patients will be based on a combination of therapy with mTORC inhibitors with secondary prophylactic treatment of hypercholesterolemia.

Besides, the impact on complex mTORC insulin also stimulates the activity of Akt kinase, which through phosphorylation modulates the activity of a number of metabolic pathways. It has been proven to be related higher activity of the glucose transporter Glut, such as 1 and 4 in cancer cells. The study found that the protein kinase Akt inactivates TSC-2 (tuberous sclerosis complex 2), which activates the GTP-ase Rheb. In this way, mTORC pathway is stimulated by Akt kinase [42–44]. The study by Sundqvist et al. demonstrated that Akt kinase by deactivation of kinase 3 of glycogen synthase (GSK3) enhances SREBP proteins functioning. Its result is an increased activity of LDLR and guideboards of cholesterol in the cells [45, 46].

Liu et al. proved that the inflammation induced by IL-1β was the result of the activity trail of mTORC and its effect on SREBP-2 and the expression of LDLR. Administration of rapamycin reversed this effect by increasing the expression of PCSK9 [47]. MTORC pathway inhibitors have been used as a component in a new generation of coronary stents. Clinical studies confirmed that their use reduced the number of thromboses and restenoses, thus reducing the need for repeated coronary intervention [48].

Most PCSK9 is synthesized in the liver. In much smaller quantities, it is expressed in the kidney, adrenals, pancreas, small intestine, and brain, as well as in the atherosclerotic plaque foam cells. It is known that atherosclerosis is an inflammatory process, which involves multiple cytokines. In Tang et al. research, macrophages stimulated by OxLDL derived from THP-1 cells (human leukemic cell line 1) synthesized increased amounts of IL-1α, IL-6, TNF-α, and also PCSK9. This process was dependent on the transcription factor NF-kB (nuclear factor kappa-light-chain-enhancer of activated B cells). OxLDL stimulation resulted in the degradation of proteins inhibiting the translocation of transcription factor (IκBα) into the nucleus and its activation. PSCK9 inhibition by small interfering RNA (siRNA) had anti-inflammatory properties and blocked the function of NF-kB [49]. PCSK9 level also correlated with the level of leukocytes, CRP, and fibrinogen in patients with stable coronary disease [50].

The research by Ruscic et al. demonstrated an effect of TNF-α on JAK/STAT pathway, whose negative regulator is SOCS3 (suppressor of cytokine signaling 3). The increased expression of SOCS3 caused inhibition of STAT3 (signal transducer and activator of transcription proteins 3) phosphorylation and therefore increased expression of PCSK9. The study also found an increased level of enzymes involved in lipogenesis: FAS (fatty acid synthase) and SCD-1 (stearoyl-CoA desaturase 1). Furthermore, activation of SREBP-1c and accumulation of triglycerides and ApoB was also noted. Similar properties to TNF-α on JAK/STAT pathway were proven for insulin, which in a SOCS3-dependent way stimulated the synthesis of PCSK9 [51]. The experiment led by Cao et al. revealed that the administration of oncostatin M (OM—belonging to IL-6 family) to HepG2 cells affected the increase of LDL receptor expression, while at the same time, decreased the expression of PCSK9 in a way dependent on kinase pathway of JAK1 and JAK2 and MEK1 (mitogen-activated protein kinase kinase 1)/ERK (extracellular signal-regulated kinases) [52].

The primary role in the process of changes taking place inside the plaque, which lead to rupture and thrombogenicity growth, plays composition and not size. Hypercholesterolemia seems to have secondary importance. Giunzoni et al. showed that PCSK9 may mediate the inflammatory process in atherosclerotic plaque by increased infiltration of inflammatory monocytes and their differentiation to macrophages. Administration of LPS induced in macrophages a growth in the expression of pro-inflammatory cytokines IL-1β and TNF and inhibition in the expression of inflammatory markers Arg1 and IL-10. It was also found that the pro-inflammatory activity of PCSK9 in the atherosclerotic plaque is dependent on the presence of LDL receptor [53].

Increased turnover of LDL may play a role in the defense of human body against pathogens. Bacterial lipids may be transported involving HDL, LDL, and VLDL and be subject to circulation similar to cholesterol. Mice deprived of PCSK9 synthesized smaller amounts of IL-6, TNF-α, MCP-1 (monocyte chemotactic protein 1), and MIP-2 (macrophage inflammatory protein 2) in response to stimulation by LPS. Moreover, smaller drops of temperature, pressure, and ventricular ejection of the left heart fraction were reported in their bodies. Six hours after the administration of endotoxin, its level was lower by 55% in comparison to the control group. Patients with septic shock with LOF PCSK9 mutations had greater survival rate as compared to patients with GOF mutations after 28 days of observation (71.8% compared to 56.8%) [54]. Experiments point to new opportunities of PCSK9 inhibitors in the regulation of inflammation, not only in the atheromatous plaque but also during sepsis.

## PCSK9 and apoptotic pathways

**Fig. 1:**
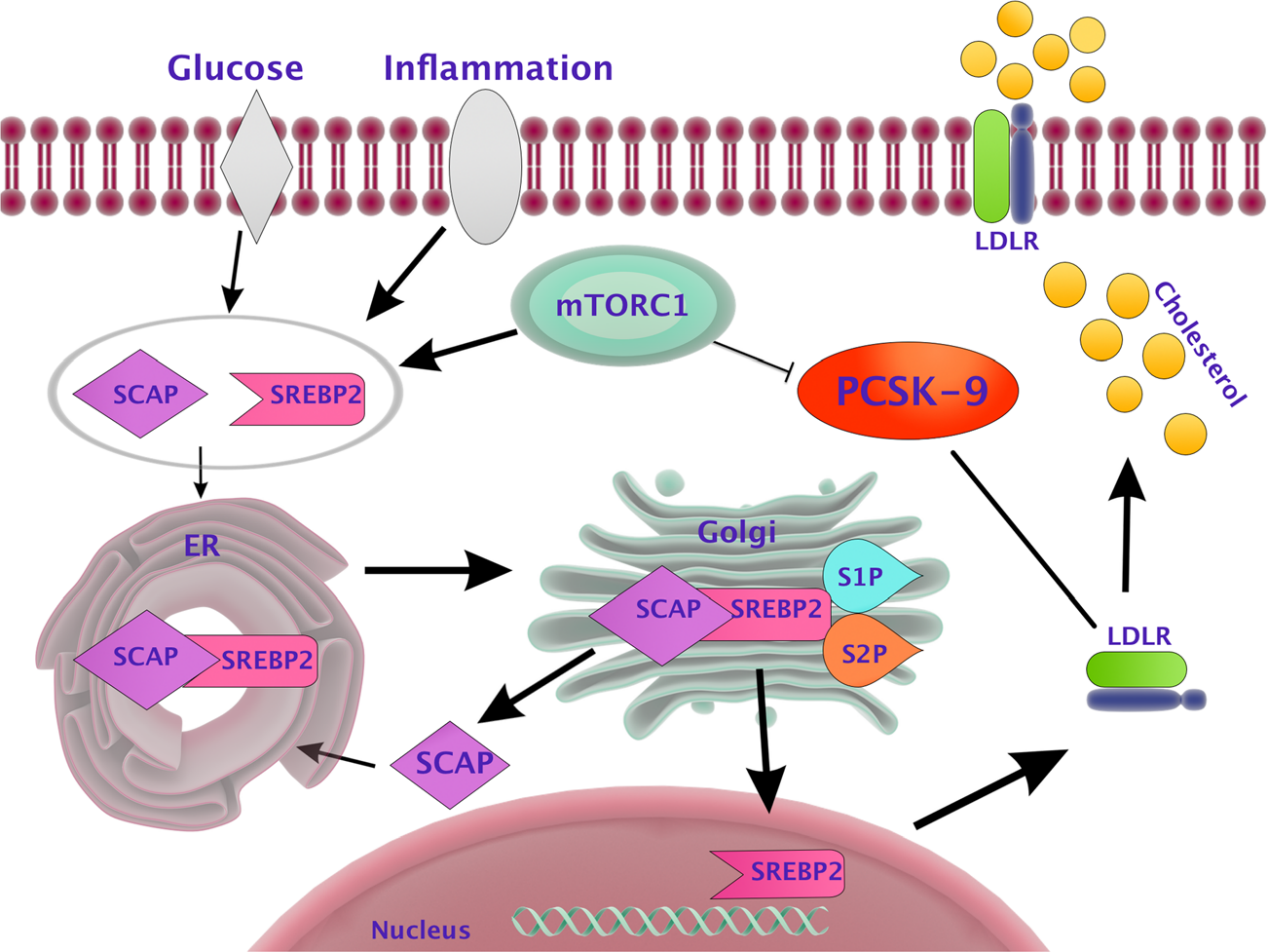
PCSK9 in inflammatory and diabetic pathways. This figure is based on [46]. PCSK9 synthesis is inhibited by TORC complex that can be influenced by pro-inflammatory cytokines (IL-6, TNF) or hyperglycaemia. In ER after separation of INSIG protein, SCAP/SREBP-2 complex is activated and subsequently matures in Golgi apparatus under the influence of two proteases: SIP-1 and SIP-2. In the next phase, nuclear transcripting factor SRBP2 permeates into the nucleus where it reacts with LDLR gene promoter (SRE-1) enhancing its transcription. This results in a synthesis of LDLR receptor and increased LDL absorption on the hepatocyte surface. *mTORC* mammalian target of rapamycin complex, *TNF* tumor necrosis factor, *ER* endoplasmic reticulum, *INSIG* insulin induced gene, *SCAP* SREBP cleavage-activating protein, *SREBP* super conserved receptor expressed in brain, *SRE* serum response factor, *LDLR* LDL receptor

The apoptosis ability of cells is described in oncology by the ratio of Bax/Bcl-2. Bax is the protein that accelerates apoptosis by forming pores in the outer membrane of mitochondria and increasing its permeability. Bcl-2 has the opposite effect, that is, it inhibits apoptosis. In their experiment, CY et al. disclosed the correlation between increased vascular endothelial cell apoptosis induced by oxLDL stimulation and the overexpression of PCSK9. Inhibition of the PCSK9 expression influenced changes in Bcl-2/Bax ratio and decreased the activity of caspase 9 and 3 leading to reduction in apoptosis process. Reduction of PCSK9 transcription correlated with an increased expression of ApoE2 receptor and cells lacking the gene for this receptor lost the capacity of PCSK9-dependent apoptosis (Fig. 1) [55, 56].

**Fig. 2:**
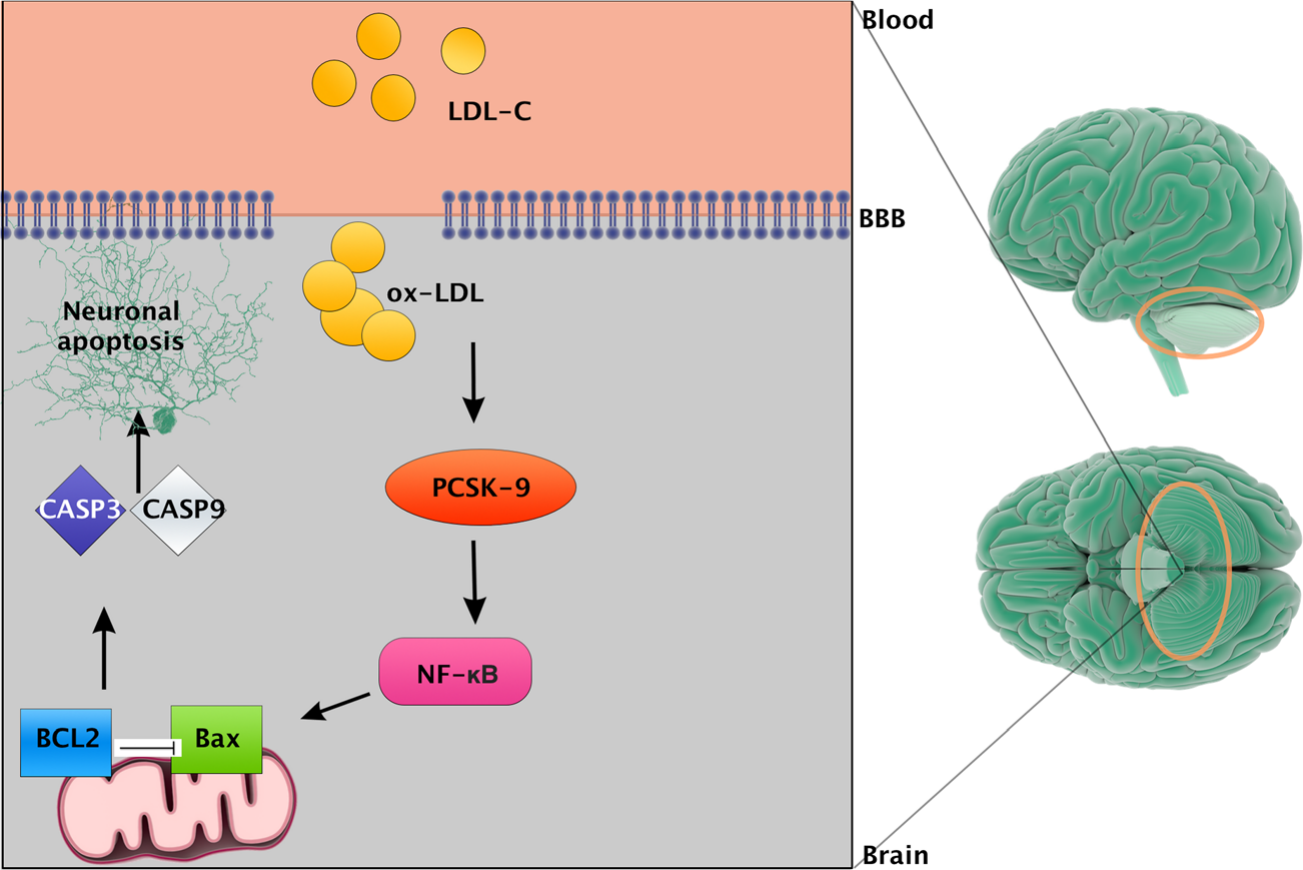
PCSK9 in neuronal apoptosis. Figure is based on [56]. In the brain, hypercholesterolemia causes the emergence of reactive forms of oxLDL which stimulate PSCK9 synthesis. Under the influence of PCSK9 an NF-κB is activated through the activation of proapoptotic Bax proteins and caspases 3 and 9. This pathway leads to neuronal apoptosis. *BBB* brain blood barrier, *NF-κB* nuclear factor kappa-light-chain-enhancer of activated B cells

The work on neuroapoptosis of cerebellar granule cells (CGN-cerebellar granule neurons) found that PCSK9 regulates neuronal death pathways involving dependent receptor ApoE2, such as JNK kinase pathway. Inhibition of PCSK9 exerts anti-apoptotic effect on the CGN cells. The study demonstrated reduction in concentration levels of phosphorylated, that is active, form of c-Jun and caspase 3. At the same time, an increased number of receptor ApoE2, independent of the VLDL receptor, was recorded. PCSK9 by influencing the degradation of ApoE2 receptor becomes a factor participating in JNK pathway and PI3K and ERK1/2 activity. Kysenius et al. found no relationship between PCSK9 and NMDA receptor (N-methyl-D-aspartate receptor) in CGN cells; however, they proved that PCSK9 is involved in the regulation of staurosporine-dependent apoptosis in those cells. In their research, inactivation of PCSK9 decreased the level of axons degradation (DRGN—dorsal root ganglion neuron) depending on the neural growth factor pathway (NGF—neuron growth factor) (Fig. 2) [57].

In the work by Piao et al. the cell of U251 brain glioma deprived of active PCSK9 gene also by the activation of caspase-3 pathway and reduced expression of oncogenes XIAP (X-linked inhibitor of apoptosis protein) and p-Akt activated apoptosis. PCSK9 also took part in the redistribution of cytochrome from mitochondria into the cytoplasm—a process initiating the apoptotic pathway. In a test, an over six times increase in the ratio of Bax/Bcl-2 was discovered, and consequently, a doubled or tripled increase in cytochrome C in cytosol. PCSK9 overexpression inhibited apoptosis in glioma cells [58].

In one of the experiments, mice fed with standard fodder lacking the gene for PCSK9 had a double lower level of melanoma metastases in the liver. Meanwhile, the high-fat diet resulted in an increase in the number of liver metastases. According to the authors, the lack of PCSK9 intensified apoptosis in hepatocytes by the increased expression of TNF mRNA and its receptor (TNFR1). TNF-α after combining with its receptor activated NF-kB, which stimulated the synthesis of anti-apoptotic proteins: Bcl-2 and TRAF-2. Hepatocytes lacking PCSK9 gene presented a reduced level of oncogenes, leading to rapid cell death [59]. The studies mentioned above lead to a promising conclusion that PCSK9 inhibitors may participate in the life span of cells by influencing apoptotic pathways.

## Clinical studies of PCSK9 inhibitors

PCSK9 inhibitors are monoclonal antibodies connecting to PCSK9 protein and blocking its activity. Deactivated form of PCSK9 cannot carry out its function, which consists in the promotion of LDL receptor degradation. There can be distinguished two human monoclonal antibodies: alirocumab and evolocumab, of IgG1 class and one humanized mouse monoclonal antibody of IgG2 class, bocacizumab.

PCSK9 inhibitors are registered in Europe for treatment in primary hypercholesterolemia (heterozygous familial and non-familial) or, as an adjunct to diet, in mixed dyslipidemia. They are administered in combination with a statin solely or a statin with other lipid-lowering therapies for patients unable to reach LDL-C targets, either with the maximum tolerated statin dose or in combination with other lipid-lowering therapies for statin-intolerant patients.

So far, there have been three meta-analyses worldwide of the new group of drugs in patients treated with high doses of statins or ezetimibe. In Zang’s et al. meta-analysis of 25 randomized control trials (RCT) encompassing a group of 12,200 patients treated with evolocumabe and alirocumab throughout the period of 12 weeks, a decrease in LDL concentration by 60.4% for evolocumabe administered every 2 weeks at a dose 140 mg in comparison with placebo was recorded. In the mentioned meta-analysis, LDL decreased on average by 81.6 mg/dL for evolocumabe. A slight HDL increase by 6.9 and 7.2% was recorded in comparison to placebo and ezetimibe.

The results for alirocumab were similar. LDL concentration decreased on average by 52.6% in comparison to placebo. HDL-C increased by 8%. Values of remaining lipoprotein fractions, such as TC, non-HDL-C, VLDL-C, ApoB, TG, and Lp(a), also decreased significantly (by −40.48, −56.07, −24.83, −52.69, 17.35, and −32.39%, respectively). The meta-analysis also determined a decrease in the all-cause mortality rate by 57%. Both PCSK9 inhibitors turned out to be safe. Injection-site reactions were the most common (relative risk 1.48, 95% CI 1.5–2.09, *P* = 0.02). The incidence of treatment-emergent adverse events (TEATs) during evolocumabe administration was 52.2 versus 45.2% in the placebo group. In the alirocumab group, the occurrence of neurocognitive impairments was 0.6%. [60].

In the second meta-analysis by Navarese et al. encompassing 24 RCT on a group of 10,000 patients treated with PCSK9 inhibitors, it was determined that LDL decreased by 58.7% as compared to placebo. HDL-C increased by 6.3%. The population taking new drugs had a lower all-cause mortality rate by 41.5% in comparison to the placebo group. What is more, a statistically significant difference in the myocardial infarction works to the advantage of the new drugs (OR 0.49, 95% CI 0.26–0.93, *P* = 0.03) [61].

In the third meta-analysis of 17 randomized controlled trials including 13,000 patients with primary hypercholesterolemia receiving PCSK9 inhibitors recorded a reduction of LDL-C level by 57%, total cholesterol by 46%, apo B by 53%, and PSCK9 by 59%. The level of HDL and apolipoprotein A1 increased in the studied group. Administration of new drugs resulted in a 57% reduction in all-cause mortality. Also, there was a visible trend towards a reduced mortality rate related to cardiovascular disease and a reduced incidence of cardiovascular events. Patients treated with inhibitors had five times lower level of creatine kinase and three times lower level of liver transaminases compared to placebo. Unfortunately, the study documented a greater than doubled increase in the incidence of cognitive impairment [62].

Astonishing outcomes of the meta-analysis in reducing cardiovascular incidents can result from atherosclerotic plaque regression. In GLAGOV study, patients suffering from cardiovascular disease treated with evolocumab for 18 months had −1% percentage atheroma volume (PAV) reduction assessed by IVUS (intravascular ultrasound). Total atheroma volume (TAV) had dropped to 5.8 mm^3^ as compared to placebo 0.9 mm^3^. Evolocumab reduced LDL from baseline value 93 to 36.6 mg/dL. 65.7% of patients had plaque regression. What is interesting, patients whose LDL had been lower than 70 mg/dL achieved even better results. Their PAV was −1.97% and 81% of patients had plaque regression. In this group, average LDL amounted to 24 mg/dL. What level of LDL is safe? Can we reverse atherosclerosis by PCSK9 inhibitors? These questions should soon be answered [63].

In March 2017, FOURIER study results were released pertaining to evolocumabe administration once a month at the dose 420 mg or every 2 weeks at 140 mg to a group of patients receiving from moderate to high doses of statins. It was a randomized, double-blinded, placebo-controlled clinical trial of 27,564 patients suffering from cardiovascular diseases, such as myocardial infarction, stroke, or peripheral artery disease. The observation period lasted on average 2.2 year. The administration of PCSK9 inhibitor decreased the risk of primary endpoint occurrence, that is, mortality due to cardiovascular disease, myocardial infarction, stroke, hospitalization for unstable angina, and percutaneous coronary intervention by 15%. Secondary endpoints, such as relative risk of death due to cardiovascular disease, were decreased by 20%, myocardial infarction by 27%, stroke by 21%, and repeated revascularization by 22%. Evolocumabe reduced LDL concentration by 59% from *z* 92 to 30 mg/dL. Eighty-two percent of patients reached an LDL level below 70 mg/dL, and 42% of the studied group registered an LDL-C quantity below 25 mg/dL. The study did not record any statistically significant side effects of the drugs and especially not any neurocognitive side effects, which was feared due to the outcomes of past research [64].

It is known that monoclonal antibodies against PCSK9 are not able to overcome the blood-brain barrier. They can only influence peripheral nerves. There was no dysfunction in the functioning of the peripheral nervous system in studies on safety of usage of PCSK9 inhibitors in animals [65]. Carriers of LOF (loss-of-function) mutations in PCSK9 with very low LDL cholesterol levels do not show mental and physical disorders [66–68].

Most data on neurocognitive disorders was presented by EBBINGHAUS study. In that research, 2000 FOURIER study patients underwent various tests evaluating their neurocognitive abilities within the period of 20 months. It was not concluded that new drugs deteriorated memory, logical thinking, or caused headache. Again, injection side reactions were the most common. They occurred in 2.1% patients in the evolucumabe group and in 1.6% patients in the placebo group. Allergic reactions occurred in 3.1% patients and in 2.9% of the control group. Only in 0.3% patients evolocumabe antibodies were recorded. Neutralizing antibodies, that is, antibodies that compete with monoclonal antibody to bind PCSK9 protein, were not determined at all.

For comparison’s sake, it is worth to present the outcomes of immunogenicity for alirocumabe from ten clinical studies conducted on 4747 patients. The follow-up lasted up to a maximum of 78 weeks. Antibodies against the drug were recorded in 5.1% patients of the PCSK9 inhibitor group and in 1% of the placebo group (a false-positive outcome). During the study, no influence of antibodies on final LDL concentration was recorded. Neutralizing antibodies that emerged in 1.3% patients also did not modify the low LDL level whereas in patients with antibodies against the drug, a higher frequency of injection side reactions was observed [69].

Even more interesting data on PCSK9 inhibitors immunogenicity was delivered by prematurely ended SPIRE-1 and SPIRE-2 studies that included together over 28,000 patients. Those trials estimated the influence of bococizumab on the incidence of cardiovascular events and its safety. Bococizumab, in contrast to evolocumabe and alirocumabe, is not a fully human humanized antibody but partially mouse monoclonal antibody. Those studies recorded the occurrence of antibodies against the drug in 48% patients and neutralizing antibodies in 29% patients. The presence of antibodies against bococizumab and neutralizing antibodies caused that the reduction of LDL concentration by 54.2% as recorded in the 12th week of the study started to gradually decrease to 43% in the 52nd research week. In conclusion, it has been established that the higher the level of antibodies against the drug rose, the lower LDL levels dropped. Additionally, it has been observed that patients were characterized by a huge volatility of LDL concentration during the trial, independent of the presence of antibodies against the drug.

In the SPIRE-1 study with follow-up of 7 months, which included 16,817 patients with LDL >70mg/dl, there was no benefit with respect to major adverse cardiovascular events. On the other hand, in the SPIRE-2 study, a group of 10,621 patients of LDL >100 mg/dL benefited from the administration of bovacizumabe, as the primary end point dropped by 21% [70].

The biggest shortcoming of PCSK9 inhibitors is their high price. An annual cost of evolocumabe and arilocumabe treatment in the USA totals to over 14,000 dollars. In the FOURIER research, the number need to treat (NNT) equaled 74. Precisely, this number of patients needed to be treated for 2 years of the study duration to prevent one of the primary end points, which meant an expense of over 2 million dollars. Cost-effective analyses indicate that current price needs to be reduced by 85% to make PCSK9 inhibitors viable [71].

## PCSK9 synthesis blocking drugs

Great expectations are placed within a new group of drugs whose activity is based on a siRNA that is located in the lipidoid nanoparticle (LNP) and inhibits the translation of mRNA PCSK9. Inclisiran is a representative of this group. This compound is built of two combined RNA strands. One of the strand is called guide. Its role is to connect PCSK9 protein with an appropriate, protein coding mRNA fragment. The second strand, called passenger, is responsible for the process of connecting to RNA-induced silencing complex (ROSC) in which the translation of PCSK9 mRNA is inhibited. Inclisiran underwent various modifications which made it stronger and more stable. Long activity of the drug ensures resistance to the egzo- and endonucleases. Changes to the composition of the drug eliminated immunogenicity, which in the light of recent reports on bococizumab, is very encouraging. The implemented adjustments increased receptor characteristic by adding GaINC fragment which enables combining with asialoglycoprotein receptor (ASGPR), which specific for liver cells. PCSK9 mRNA is cleaved after connecting to ROSC [72]. Inhibiting PCSK9 synthesis leads to an increase of LDL receptors on the surface of hepatocytes and in this way contributes to decreasing of LDL particle concentration in blood plasma.

Inclisiran effectiveness was confirmed in a second phase study ORION-1. Five hundred one patients with cardiovascular diseases and high level of cholesterol who were treated with statins were qualified for this study. Participants were divided into eight groups which were administered the drug at the dose of 100, 200, 300, and 500 mg and placebo, respectively. The primary end point was the reduction of LDL concentration on the 180th observation day. The optimum scheme turned out to be drug administration of 300 mg in two doses, which in the 180th observation day lowered LDL level on average by 52.6%. The above data suggest the possibility of administering the drug every half a year. In the 30th observation day, the lowest PCSK9 concentration was recorded reaching a level lower than 66–74% of the initial value. Eighty four of patients who received the new drug displayed LDL <50 mg/dL. Adverse reactions were present in 11% of patients. Injection side reactions were the most common. They occurred in 5% of patients. The study did not record any presence of antibodies against the drug [73].

New drugs may compete with PCSK9 inhibitors in the nearest future. High specificity towards liver cells and marginal number of side effects speak for inclisiran. In comparison to other PCSK9 inhibitors administered every month or statins administered daily, new drugs solve the problem of patient adherence to therapy. Most probably treatment cost will also be lower than that of PCSK9 inhibitors. Commencing three-phase study, ORION-4 will answer the question if inclisiran decreases the risk of cardiovascular incidents and general mortality.

## Small peptides imitating EGF-A domain of LDL receptor

Small peptides which imitate EGF-A domain of LDL receptor, that is, the location of PCSK9 binding may become a new strategy for hypercholesterolemia treatment. PCSK9 binding with small peptides through competitive inhibition with EGF-A domain of LDL receptor is supposed to inhibit receptor degradation for LDL and increase LDL particle absorption. Pep2–8 is an example of small peptides. It comprises of 13 amino acids and it is the smallest particle that inhibits PCSK9 function. Pep2–8 binding location displays a huge similarity in its structure to EGF-A LDLR domain. Pep2–8 completely blocked the binding of PCSK9 with EGF-A LDLR in experiments carried out by Zhang et al. [74]. Studies on this peptide have become fundamental to the new group of drugs. It is likely that further modifications will only enhance the properties of these small peptides and will help to introduce them onto the market.

## Conclusions

PCSK9 signaling pathways constitute a significant challenge for molecular biology. PCSK9 appears to be the mediator of many metabolic processes. PCSK9 inhibitors seem to be the strongest ally in the war against hyperlipidemia. It appears that the success of PCSK9 inhibitors does not depend only on the effect on LDL receptor degradation. Mutual relations, PCSK9, LDLR, and circulating lipoprotein should be treated as a single signaling system. Transformation control of individual parts takes place at the transcriptional and post-translational level. Cofactors directing LDLR to lysosomal degradation are still not fully understood. As expected, the key role lies in the energetic state of the cell. Daily oscillations in the concentration levels of hormones determine the fate of cholesterol.

With aging of the body, mutations and errors in metabolism accumulate. Atherosclerosis is a lifelong inflammatory process. Previous publications showed that inhibition of PCSK9 can exert anti-inflammatory effects. PCSK9 inhibitors apart from obvious ability to reduce LDL can inhibit the progression of atherosclerosis by modifying the profile of pro-inflammatory cytokines, adhesion factors, and regulatory proteins. Finally, we have evidence from clinical trial that they can reduce atheroma plaque.

PCSK9 also takes part in the regulation of apoptosis. Its increased expression through the influence on Brc-2/Bax pathway induced cell death of endothelial cells, brain glioblastoma, and increased the number of metastasis of melanoma to liver. On the other hand, blocking of the activity delayed the apoptosis of CGN cells and axonal degeneration in a manner dependent on JNK pathway and NGF, respectively. The hypothesis of anti-carcinogenic activity of new drugs is very tempting yet it requires further research.

There is a growing number of people suffering from diabetes which first symptoms are only visible in the pathology of insulin pathways. Insulin is able to stimulate mTORC complex, Akt kinase, JAK/STAT pathway, and many others. Do synthesized monoclonal antibodies modify the mechanisms of insulin resistance so that we could call them anti-diabetic? For now, there is still a lack of clear data about the effect of PCSK9 on pathomechanisms of diabetes.

There is scientific evidence on the usefulness of the new drugs to treat patients in a state of septic shock. PCSK9 inhibitors did let the temperature drop below 32 °C, increased left ventricular ejection fraction of the heart, and lead to a longer survival rate for patients in intensive care. On the other hand, it has to be born in mind that more LDL receptors mean a risk of higher vulnerability to penetration of certain viruses.

Outcomes from other studies confirm that the reduction of LDL brings a decreased general mortality and lowered risk of cardiovascular events. Blocking of PCSK9 protein activity has become the main goal for drugs of the new generation. The majority of data supports administration of PCSK9 inhibitors; however, it has to be bore in mind that the longest observations in this area have only just passed 2 years. The studied drugs display little adverse effects in comparison to statins, yet treatment costs remain disproportionate to the achieved benefits. New strategy of treatment that is based on the inhibition of PCSK9 mRNA is very promising. The greatest advantage of inclisiran will certainly be the possibility of administering it twice a year and in this way diminishing the rate of adverse reaction related to drug injection. It is possible that the new competitor of PCSK9 inhibitors could cost less. Yet, any premature applause should be constrained for now, as knowledge gained so far is based solely on one study of the second phase, which involved only 500 patients. Without a doubt, the idea of introducing mini peptides imitating EGFA-A domain of LDL receptor constitutes an even more interesting perspective. Thus, it cannot be ruled out that one day in future, the smallest one will become the greatest one.

The story of PCSK9 is still evolving and constantly intriguing by its dynamics. Expectations of PCSK9 inhibitors are high and well justified by previous discoveries.

ARH, low-density lipoprotein receptor adapter protein 1; CETP, cholesteryl ester transfer protein; CHRD, C-terminal domain rich in cysteine and histidine; COP, coated protein complex II; EGF, epidermal growth factor; ERK, extracellular signal–regulated kinases; GOF, gain-of-function mutation; H1NFP, histone 1 nuclear factor protein; HNF, hepatocyte nuclear factor; IL, interleukin; INSIG, insulin-induced gene; JAK, janus kinase; LOF, loss-of-function mutation; LPS, lipopolysaccharides; LRP1, low-density lipoprotein receptor-related protein 1; LXR, liver X receptor; MCP, onocyte chemotactic protein 1; MEK1, mitogen-activated protein kinase kinase 1; MIP-2, macrophage inflammatory protein 2; mTORC, mammalian target of rapamycin complex; NF-κB, nuclear factor kappa-light-chain-enhancer of activated B cells; NMDA receptor, N-methyl-D-aspartate receptor; NPC1L1, Niemann-Pick C1-like 1 protein; PCK1, phosphoenolpyruvate carboxykinase 1; PCSK9, proprotein convertase subtilisin/kexin type 9; PI3K, phosphatidylinositol-3-kinases; SCAP, SREBP cleavage-activating protein; SCD-1, stearoyl-CoA desaturase 1; SOCS3, suppressor of cytokine signaling 3; SRE, serum response factor; SREBP, super conserved receptor expressed in brain; SSD, sterol-sensing domain; STAT, signal transducer and activator of transcription proteins; THP1, human leukemic cell line 1; TRAF2, TNF receptor-associated factor 2; XIAP, X-linked inhibitor of apoptosis protein.
